# Strategies for the Design of PEDOT Analogues Unraveled: the Use of Chalcogen Bonds and σ-Holes

**DOI:** 10.1021/acs.jpca.2c08965

**Published:** 2023-04-19

**Authors:** Dominik Farka, Kristian Kříž, Jindřich Fanfrlík

**Affiliations:** Institute of Organic Chemistry and Biochemistry, Czech Academy of Sciences, Flemingovo Nám. 2, 160 00 Prague, Czech Republic

## Abstract

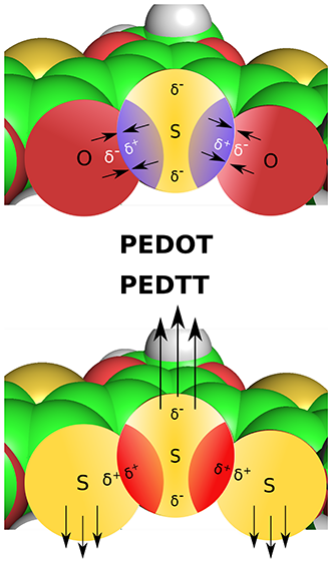

In this theoretical study, we set out to demonstrate the substitution effect of PEDOT analogues on planarity as an intrinsic indicator for electronic performance. We perform a quantum mechanical (DFT) study of PEDOT and analogous model systems and demonstrate the usefulness of the ωB97X-V functional to simulate chalcogen bonds and other noncovalent interactions. We confirm that the chalcogen bond stabilizes the planar conformation and further visualize its presence via the electrostatic potential surface. In comparison to the prevalent B3LYP, we gain 4-fold savings in computational time and simulate model systems of up to a dodecamer. Implications for design of conductive polymers can be drawn from the results, and an example for self-doped polymers is presented where modulation of the strength of the chalcogen bond plays a significant role.

## Introduction

A chalcogen bond^1,2^ is a lesser-known noncovalent interaction that belongs to the class of σ-hole interactions. These interactions are brought about by the existence of a σ-hole, an area on the atom that is relatively more electro-positive than its surroundings. This electro-positive region then enables the σ-hole displaying atoms to interact with nucleophiles.^3^ Despite being nonclassical, they are being increasingly recognized across many fields.

They have been shown to be one of the factors that determine protein structure and protein–ligand interaction.^4−8^ It has also been demonstrated that they are important for the stability of small molecular complexes,^8,9^ supramolecular complexes,^10,11^ and crystal assemblies.^11−13^ They can also facilitate catalysis,^14−16^ and their role for assembly or conformation of polymers has also been shown.^17,18^

**Figure 1:**
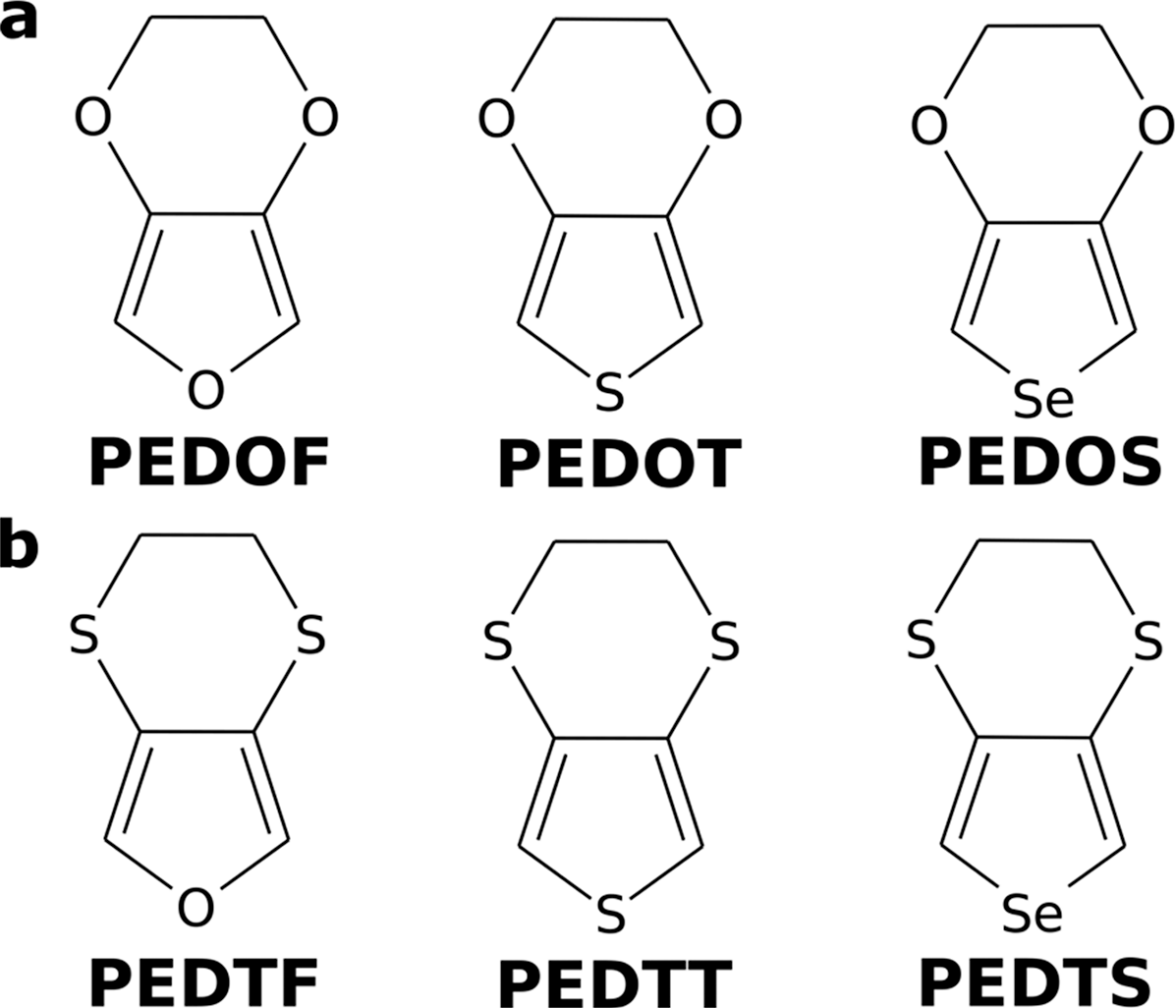
Structural formulas of the monomers constituting (a) the oxygen series (dioxane side-group) and (b) the sulfur series (thia-dioxane side group). For clarity, we chose to use the polymers abbreviations (the monomer abbreviations would lack the first letter “P”).

Poly(3,4-ethylenedioxythiophene) (PEDOT, Figure 1a) is one of the most intensively studied polymers.^19^ This polymer is mainly used in applications such as transparent lightweight electrodes, diodes, and solar cells,^20−22^ neural and artificial cellular signaling,^23,24^ or as a high-performing conductor.^25−29^ This is possibly the case due to its high performance, easy handling, and stable quality. These properties were suggested to lie in the intramolecular S–O interactions that render the polymer intrinsically planar,^30^ facilitating the conductivity via a π-orbital overlap between the monomers.^31,32^ In contrast, its all-sulfur analogue, poly(3,4-ethylenedithiathiophene) (PEDTT, Figure 1b), exhibits intramolecular S–S repulsive contacts, which distort the polymer and render it essentially insulating.^33,34^

The phenomenon of noncovalent interactions stabilizing the planar conformation was later termed “conformational locking”,^31^ and the S–O interaction in PEDOT (and similar molecules) was recognized as a chalcogen bond.^32^ However, despite these properties were attributed to chalcogen bonds, their presence was never explicitly proven computationally, say, through the electrostatic potential (EPS) surfaces.

Herein, we present a quantum mechanical study of the role of these σ-hole interactions in trimers, hexamers, and dodecamers of PEDOT, PEDTT, and their analogues (Figure 1). We investigate the substitution effects of the chalcogens (O, S, Se) on their geometry and examine their energetic stability by means of angular scans, keeping in mind that deviations from planarity hamper conjugation through a decrease of the π-orbital overlap on the carbon atoms.

We show the advantage of the ωB97X-V functional to find geometries in these systems rich in chalcogen-bonds and repulsions by comparison to calculations in B3LYP. The former allows for an up to 4-fold saving in computational time for the same number of computational cycles. This translates to tens of hours saved in the case of the dodecamer. In this way, we set an example for further studies in polythiophenes.

This publication serves as a complement to experimental work in PEDOT-like conductors, especially for emerging self-doped systems where the chalcogen-bond can be inadvertently weakened by substitution effects.^35^ In that we present an answer to a mechanism of how to establish planarity in an intrinsically distorted polymer such as PEDTT.^36^

## Methods

### Model Systems and Optimization

The trimer model systems are informative, because, first, they serve as the smallest unit that represents all geometric features found in the polymer (Figure 4).^37^ Moreover, conductive polymers are rendered conductive via doping, where one dopant associates with at least three monomer subunits.^19,36^ However, longer chains are necessary to improve the overlap of predictions with experiment.^38^

Therefore, we have also prepared larger model systems (hexamers and dodecamers, further referred to as “oligomers”) to investigate how the systems properties change with increasing size (for instance, due to extended conjugation).

We limit ourselves to the investigation of a single polymer chain in this manuscript.

For the corresponding analogues, we took PEDOT and PEDTT oligomers as starting points and then substituted the chalcogen atom in the thiophene ring. The substitution by oxygen resulted in furan, and the substitution by selenium resulted in selenophene. Hence, we acquired two separate series of molecules: PEDOF, PEDOT, and PEDOS “oxygen series”, Figure 1a, and PEDTF, PEDTT, and PEDTS “sulfur series”, Figure 1b.

All model systems investigated herein were optimized in TURBOMOLE 7.3^39^ with the B3YLP using the DZVP-DFT basis^40^ and D3 dispersion correction.^41^ Where relevant for subsequent investigation, the ωB97X-V functional and DZVP-DFT basis were used.^40^ The ωB97X-V was previously demonstrated to be excellently suited for investigation of σ-hole related interactions.^42,43^ Further, range-separated functionals such as ωB97XD have been previously demonstrated suitable for the simulation of conductive polymers.^38,44^

For all the TURBOMOLE calculations, including angular scans (see next section), the cuby4 interface program^45^ was used. The interface supplied the D3 empirical dispersion with Becke-Johnson (BJ) damping^46^ (used for B3LYP calculations) and handled the quasi-newton (BFGS) optimization procedure (used for both functionals).^45,47^

For better coverage of conformational space, we performed the optimizations from multiple starting points defined by different dihedral angles between monomers (Figure 4, D_*x*_ values)

The relevant bond lengths and noncovalently interacting atoms of optimized trimers were analyzed.

The energy levels were calculated at the same parameters as the geometry scans using the ωB97X-V functional with the following exception. All calculations were achieved with the lbfgs optimizer, spin-unrestricted, and the self-doped doublet was calculated in its charged state and required preoptimization with a level shift of 0.4 to achieve initial convergence before reoptimization.

All geometries were visualized via Pymol software.^48^

### Electrostatic Potentials

To visualize the σ-holes, for each structure of the oxygen series, we created model systems in which we rotated one monomer-subunit by 90°. This way the electronic densities at sites of interest do not merge together, allowing better examination of the phenomenon in question.

The electrostatic potentials (ESP) were calculated at the HF level^49^ with the def2-QZVP basis set, using *Gaussian* 09.^50^ The ESP map was constructed at the 0.001 electrons per Bohr^3^ density isosurface.

### Angular Scans

To examine the effect of the deplanarization on the stability of the planar conformation, we perform “angular scans”. We chose the center-most monomer–monomer bond (see Figure 4, D_1_, for the trimer) of the optimized geometries and alter their dihedral angle; thus, we established the following series with “0°” marking the planar trans-conformation: 0, 15, 30, 45, 60, 90, 120, 150, and 180°.^51^

This was repeated for all oligomers to observe chain-length effects. The energy of each of the resulting points was computed with the ωB97X-V functional and the bigger def2-QZVP basis set.^52^ This functional has been previously shown to be particularly suited for the simulation of repulsions and chalcogen bonds.^42,43^

The results were recalculated using the B3LYP functional. For the two longer oligomers, only 0, 60, 90, and 120° were addressed to limit the computational effort.

The energetic minimum was taken as a point of reference to visualize the energy penalty of each corresponding rotation.

The band gaps for respective scan points were estimated as HOMO–LUMO differences, which were provided by the same computation.^53^

## Results and Discussion

It has been shown that the σ-hole becomes increasingly positive with increasing atomic weight of the element in question. Conversely, lighter elements, like second row nitrogen or oxygen, exhibit no appreciable σ-hole,^1^ save rare exceptions.^54^

Thus, as it has been proposed before,^30,32^ σ-holes on heavier chalcogens would interact with electronegative dioxane oxygens (for PEDOT, see Figure 4, C1–C4 distances) via a chalcogen bond, resulting in conformational locking^31^ and stabilization of the planar conformers.

**Figure 2:**
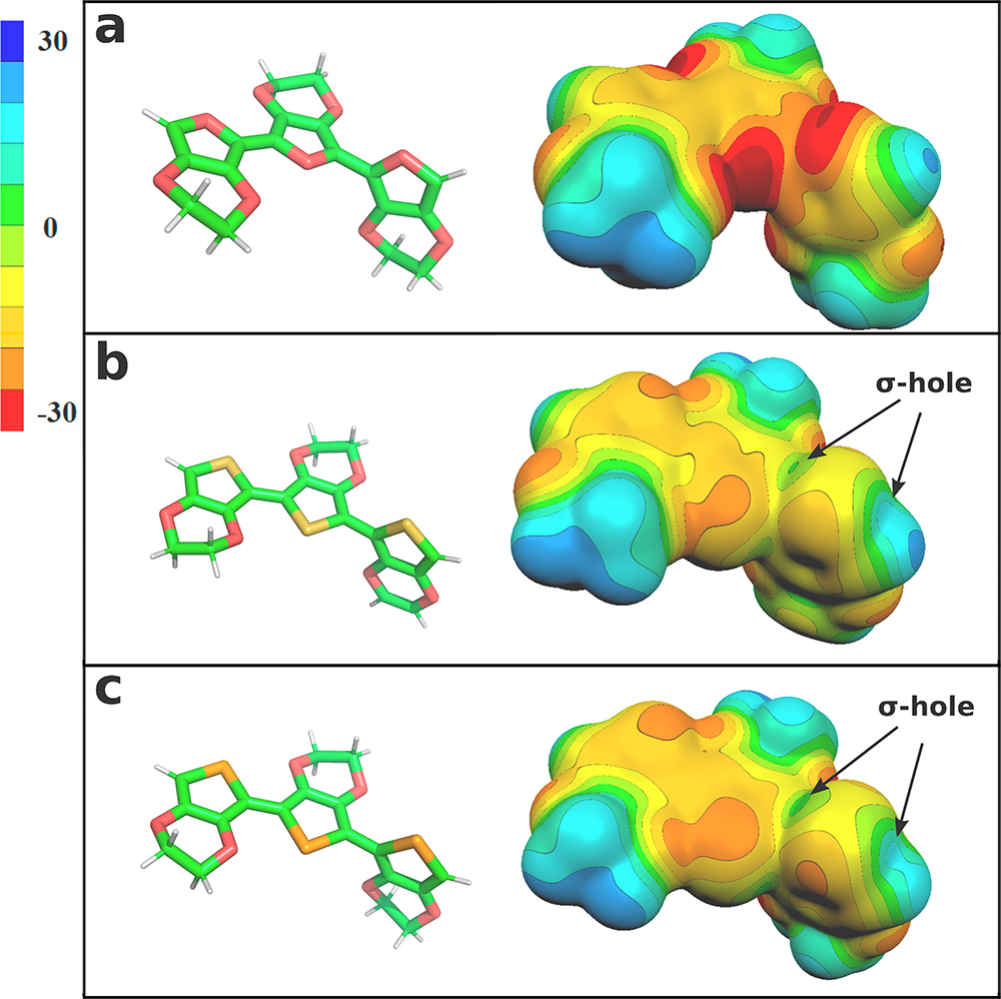
Electrostatic potential molecular surfaces of (a) PEDOF, (b) PEDOT, and (c) PEDOS. Constructed at 0.001 electrons per Bohr^3^ isosurface. One monomer is rotated 90° from the molecular plane.

**Figure 3:**
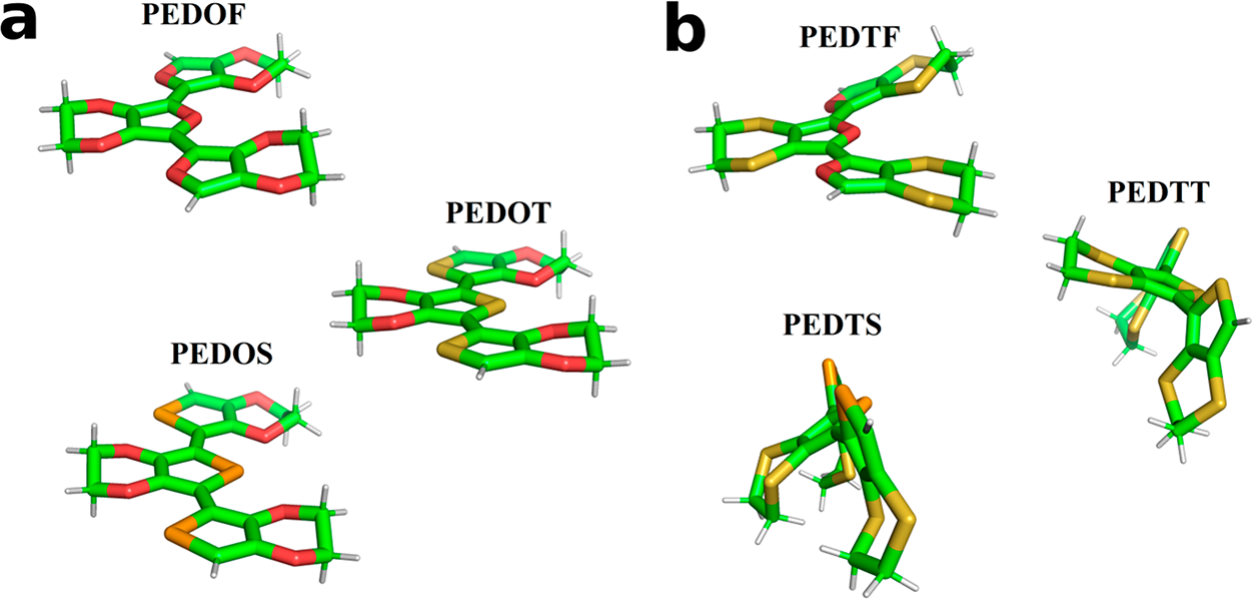
Molecular structures of trimers in their energy-minima. All three PEDOT analogues of oxygen series (a) were essentially planar, while the PEDTT sulfur analogues (b) were nonplanar due to chalcogen–chalcogen repulsions, the absence of the chalcogen bond.

**Figure 4:**
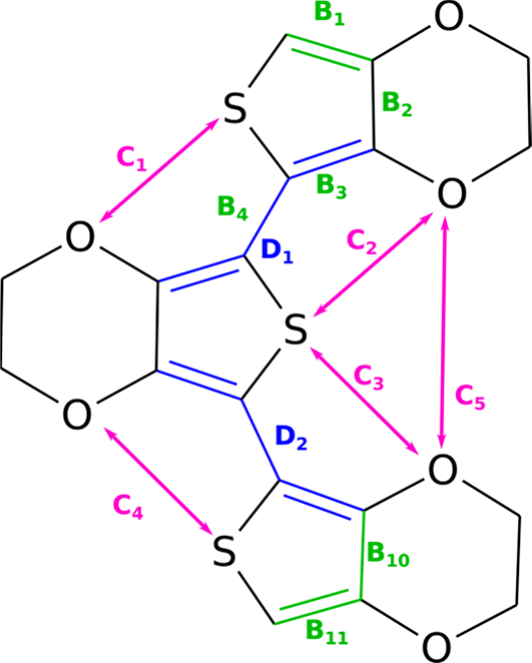
Illustration of measured geometrical quantities, where B_*x*_ designates bond lengths of the conjugated system (they appear in Table S2), D_*y*_ designates the dihedral angles, and C_*z*_ designates the chalcogen–chalcogen distances.

**Figure 5:**
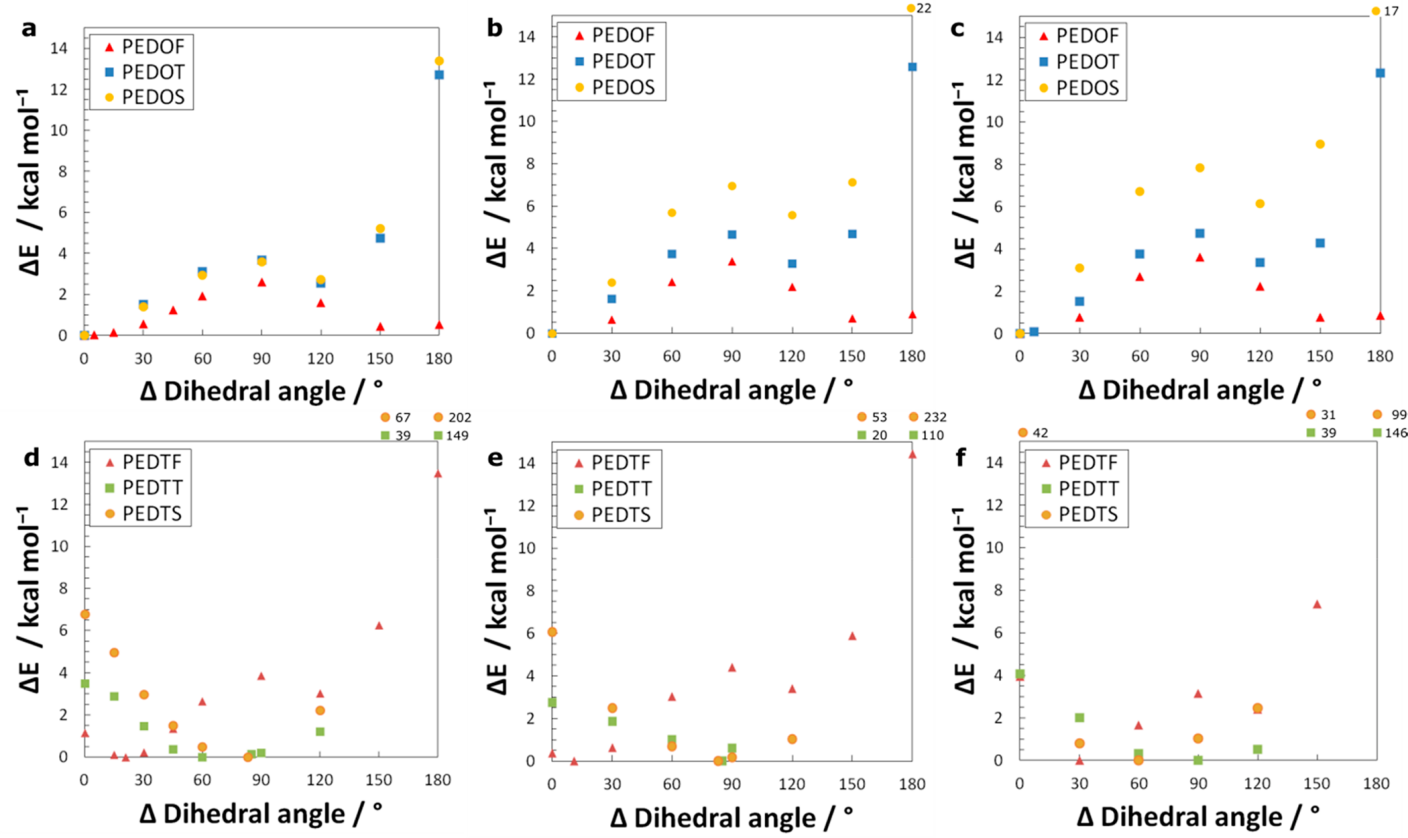
Conformational energies (Δ*E*) that are dependent on the dihedral angle are recalculated via B3LYP/D3BJ analogous to Figure 5. We present a comparison between the trimers, hexamers, and dodecamers of the oxygen (a–c) and sulfur series (d–f).

To examine this possibility, we first showed the electrostatic potentials (ESP) for model systems (Figure 2). Second, we discussed the optimization of models, searching for minima (Figures 3 and 4, Table 1; SI, Tables S1 and S2). Finally, we performed an analysis of energy and band gap variation with the rotation of a single monomer (Figure 5 and SI, Figures S2, S4, and S5).

**Table 1:** Molecular Geometries of Energy-Minima in Trimers^a^

	PEDOF	PEDOT	PEDOS	PEDTF	PEDTT	PEDTS
dihedral angle (°)						
D1	178.3	179.5	179.2	179.8	85.9	70.2
D2	176.3	178.6	179.3	158.5	81.3	66.9
chalcogen–chalcogen separation (Å)						
C1	3.07	2.95	2.93	3.05	4.02	4.57
C2	3.07	2.94	2.93	3.13	4.06	4.44
C3	3.07	2.94	2.93	3.13	4.16	4.46
C4	3.07	2.95	2.93	3.08	4.16	4.45
C1 (Pl.)	3.07	2.95	2.93	3.03	2.82	0.93
C5	4.10	5.00	5.26	3.84	7.10	6.11
C5 (Pl.)	4.10	5.02	5.26	3.49	4.24	4.93
chalcogen–chalcogen separation (% of vdW sum)						
C1–C4	101	89	86	93	114	121
C1 (Pl.)	101	89	86	91	78	79
C5	135	164	173	106	197	170
C5 (Pl.)	135	165	173	96	117	129

a See Figure 4 for the measures of the Pl. values that correspond to the forced 180° planar conformation (modification of dihedral angles after the optimization). The most stable conformers of PEDTT and PEDTS were found to be distorted structures.

### Electrostatic Potentials

While there were no apparent σ-holes for PEDOF, they were observed on the ESPs of PEDOT and PEDOS (see Figure 2b,c).

Divalent chalcogens possess two σ-holes, one at the extension of each σ-bond.^1^ It can be seen in Figure 2 that the positive regions corresponding to the two σ-holes in our model systems are unequal. The σ-hole adjacent to the hydrogen cap of the model systems is larger while less distinct, as it merges with the positive potential of the hydrogen, forming a “lobe” extending from the hydrogen.

In contrast, the “σ-hole site” facing the aromatic ring appears smaller in magnitude due to the negative potential of the ring; however, it is clearly distinct both in PEDOT and PEDOS.

Consequently, caution is required when attributing an ESP value to a σ-hole, as it is impossible to isolate the electrostatic potential of a σ-hole from the influence of the rest of the molecule.

Further, it should be noted that both the S and Se essentially form a “quadrupole”. The positive partial charges (σ-holes) are in-plane of the monomer, whereas the negative partial charges (lone pairs) point out-of-plane.

### Energy Minimum Conformers

Overall, planarity, i.e., a dihedral angle ∼ 180° (Figure 4, D_1_ and D_2_), correlates with chalcogen-chalcogen distances below the van-der-Waals (vdW) radii (Table 1), indicating a stabilizing interaction, a chalcogen bond. This is the case in PEDOT and PEDOS from the oxygen series, and arguably, also for PEDTF from the sulfur series (Figure 4).

In PEDOF, for which there are no apparent σ-holes, distortion from planarity due to electrostatic repulsion between oxygen atoms was expected. Surprisingly, the optimum for PEDOF was also found to be essentially planar (Table 1, Figure 3). A closer look at the chalcogen-chalcogen distance in PEDOF reveals that the oxygen-spacing is roughly equal to the sum of the vdW radii (Figure 4, Table 1, C1–C4). Hence, Pauli repulsion is likely not prominent in this case. It seems that the planarity is established by the formation of an extended π-system, which outweighs the effects of electrostatic repulsion between the adjacent electro-negative sites of oxygen atoms. It should be noted that we found a nonplanar local minimum for PEDOF, which was energetically very close to this planar global minimum (see Table S1 in the SI). For heavier chalcogen analogues, PEDOT and PEDOS, however, nonplanar local minima found were much further in energy (see Table S1 in the Supporting Information).

For the discussion of local minima, see Supporting Information.

In comparison to the oxygen series, Pauli repulsion between chalcogen atoms in the sulfur series was an important factor in determining the geometry, resulting in a tendency to form distorted, nonplanar geometries (Table 1 and Table S1, Figure 3b).

Even in PEDTF, where the presence of sulfur–oxygen close contacts would suggest a possible chalcogen bond, as in the case of PEDOT, only one dihedral angle was planar. The second dihedral, D_2_ (Figure 4, Table 1), angle was distorted by ∼20° to minimize the effects of repulsion between sulphurs of opposing rings (C5 measure in Table 1), which would otherwise be under the sum of van der Waals (vdW) radii, 96% of the sum, if the geometry was planar (C5 (Pl.) measure in Table 1).

For both PEDTT and PEDTS, which do not have the potential to form a chalcogen bond when planar, the global minimum was found in a profoundly distorted conformation. The chalcogen–chalcogen close contacts between the neighboring rings would be well below the sum of van der Waals radii, if the planar conformation was enforced (C1 (Pl.) measure in Table 1). However, in that case, positive σ-holes would be facing each other and there would be no favorable chalcogen interaction mitigating the repulsion (as is the case in PEDOT and PEDOS). The same is true for their negatively charged lone-pairs. Hence, planar PEDTT and PEDTS are energetically disadvantageous.

Consequently, it is favorable for the PEDTT and PEDTS subunits to rotate so that the σ-holes are facing the negative sites of neighboring aromatic ring and adjacent lone-pair (Table 1, in a manner similar to our models for EPSs, Figure 2). Interestingly, the dodecamer of PEDTT optimized into a helical structure (Figure S1).

### Angular Scans and Conformational Energy

Different forces may act upon the bulk polymer, which can lead to the distortion from planar conformation and a subsequent loss of conductivity. Consequently, we seek polymers with a distinct energy barrier in the design process. We have performed angular scans along the D_1_ dihedral angle for a more detailed insight into the potential energy changes due to the corresponding rotation.

For all monomers of the oxygen series, the global minimum was found at ∼0° (Figure 5a–c and Figure S4a–c). Both functionals were in general agreement regarding energetic trends. Since the main planar conformation of PEDOF is arguably caused by conjugation, a comparison to PEDOT and PEDOS gives an estimate of the contribution of chalcogen bonds to the stability of the system. This accounts for approximately 1 kcal mol^–1^.

When we rotate the dihedral angle away from the trans-planar conformation (0°), we encounter an energy barrier (the local maximum at 90°, Figure 5) as we break the chalcogen bonds and conjugation. Beyond that, a second, rapid increase in system energy is observed, which we attribute to repulsions between approaching chalcogens of the dioxane rings. The minimum at 120° results out of an interplay between the stabilization through improved conjugation and the mentioned repulsion.

The barrier between the global and local minimum grows with chain length and is more pronounced with PEDOS (8 kcal mol^–1^); in the dodecamer, we encounter a difference of as much as 4 kcal mol^–1^ as compared to PEDOT (4 kcal mol^–1^). Owing to the fact that the barrier high-difference was negligible in trimers, it is difficult to estimate if this growth of the barrier in oligomers is due to chalcogen bonds alone or if it is due to stronger conjugation (i.e., with the influence of the heavier chalcogen).

For the sulfur series, the situation became more complex, especially with the longer chains (Figure S4d–f). In general, both functionals agree on overall trends such as the location of barriers and minima, however, B3LYP overestimates the repulsions (Figure S4d-f). Since the minima in these systems are found in nonplanar conformations, we observe disordered systems. As a consequence, the exact position of the encountered minimum may vary, yet the general trends remain.

For all oligomers of PEDTF, the global minimum is found just shy of completely planar. At that dihedral angle, the optimal geometry for the formation of chalcogen bonds is reached. In other words, the repulsion between the opposing thiodioxane sulphurs (Figure 4, distance C_5_) outweighs the stabilizing effect of optimal conjugation. Said minimum shifts due to the specifics of the tested geometry, while the overall statement remains true: PEDTF has a strong tendency to adopt a nonplanar conformation, which is in stark contrast with its quasi-constitutional analogue, PEDOT, in which the positions of sulfur and oxygen are inverted.

Thanks to the disordered nature of the minimum found in oligomers of PEDTT and PEDTS, the prevalent trends are somewhat cryptic and masked. This leads to shifting minima and even modulation of the strength of repulsion, depending on the initial geometry.

A few things are, however, very clear. First, the repulsion in the *trans*-planar (0°) conformation is significantly lower than for the *cis*-conformation (180°). Second, the selenophene (PEDTS) shows a stronger expression of minima than in the case of thiophene (PEDTT), especially in the case of the dodecamer. This is possibly related to the repulsion between the thiadioxane groups, which is probably related to the effects of conjugation on the chalcogens.

This leads to a peculiarity in PEDTT, where in the vicinity of the minimum, the dihedral angle can change by tens of degrees with virtually no cost in energy (less than 1 kcal mol^–1^). This is remarkable and demonstrates how complex the interplay of attraction and repulsion really is in this polymer.

The repulsion to form planar PEDTT was found to be 3–4 kcal mol^–1^, irrespective of chain length, and is in good agreement with results by Wijsboom et al.^53^ (periodic B3LYP). However, our own B3LYP calculations overestimate the repulsion by a factor of 2 relative to the results of Wiijsboom et al.^53^ and our own calculations in ωB97X-V (Figure S4d–f). This value is rather low and might be overcome with a sufficiently strong influence from its surroundings, such as when a strongly interacting dopant is used. This hints toward a possible explanation for the experimental work by one of the authors.^36^

The band gaps obtained by DFT simulations are typically of qualitative character only.^38,44^ To gain insight on the accuracy of our method, we compare the band gaps found in our rotational scans with experimental values found in cyclic voltametry (CV) where available.

For the oxygen series, calculations by the ωB97X-V (Figure S2a–c) functional underestimates the band gap in the case of trimers, however, delivers a reasonable result for longer chains.^19,36^ In PEDOT, cyclic voltametry indicates a band gap of 1.5 eV,^19,53^ which matches our results for the planar hexamer or the disordered dodecamer. Our findings thus match experimental findings in a significantly more accurate manner than recent findings on the band gap of PEDOT, which were achieved by the ωB97XD functional.^38^

A fairly good match with experimental CV values is also found for PEDOS (1.4 eV).^53,55^ In all cases, a lower band gap was predicted than for PEDOT, and even qualitatively, the results are in the range of experimental values. Even here, experimental values are closest to values calculated for planar hexamers or disordered dodecamers.

For the sulfur series, PEDTF exhibited the lowest band gap and even the strongest tendency of the band gap to further decrease with the elongation of the polymer chain (Figure S2d–f). Interestingly, the lowest gaps were found in the case of the *cis*-planar form. However, as this conformation is nonphysical, this value should be handled with care. It could, however, hint at the use of this motif in more complex thiophene-based monomers.

For PEDTT, the band gap expected from experimental data would be 2.2 eV.^53,56^ This is in good agreement with values found for the 60° value found for the hexa- and dodecamers. This conformation energy-wise is close to the minimum we arrived at in our calculations and suggests that calculation and experiment are in good agreement.

The close match still holds if we compare discrete energy values of respective HOMO and LUMO with experimental values.^53^ Also here, the computational values of the planar hexamer and the distorted dodecamer were closest to experimental values. The closest match was found for the intrinsically planar polymers (PEDOT and PEDOS; Figure S3).

Since our calculations are in excellent agreement with experimental CV data, it may be proposed that this functional is particularly well-suited for this type of conjugated system. Once proven on a larger scale with a variety of systems, this method may become the new method of choice for the simulation of (unodoped) conjugated polymers of even conjugated systems in general.

On a more bold note, we may in the future even gain valuable insight on the conformation of the materials observed in CV. We will focus on PEDOT, as it is the material with the most reliable data available. First, experimental data suggest a mean chain-length of around 10–12 repeat units.^29^ Second, polymers achieved via electrochemical polymerization and electrochemical doping typically display lower conductivities, which was attributed to their disorder (or, if you prefer, to amorphous structures).^19^ Consequently, it may be argued that band gaps obtained by CV inadvertently reported values for a disordered, nonplanar polymer (hence, accurate correlation with our values for disordered dodecamers). As this correlation is found for a single material, only, we see the necessity of a much larger sample size to correlate theoretical and experimental results in this fashion.

In all our calculations we were able to confirm the expected trend that polyselenophenes do have a narrower band gap as compared to polythiophenes (Figure S2a–f).

The band gap obtained from calculations in B3LYP (Figure S5a–f) overestimate experimental values for PEDOT, PEDOS, and PEDTT. In this, the functional gives a mismatch with respect to both energies and band gaps. As expected, it also underestimates rotational barriers and the effect of extended conjugation.^51^

### Substitution Effect on the Chalcogen Bonds

Next, we present some examples of how to tune the chalcogen bonds in conductive polymers. We hypothesize that this can be achieved either by influencing the σ-hole or by influencing the σ-hole acceptor. The latter is especially of interest for self-doped systems, where the doping-agent is covalently bound to the molecule of choice.^35^

**Figure 6:**
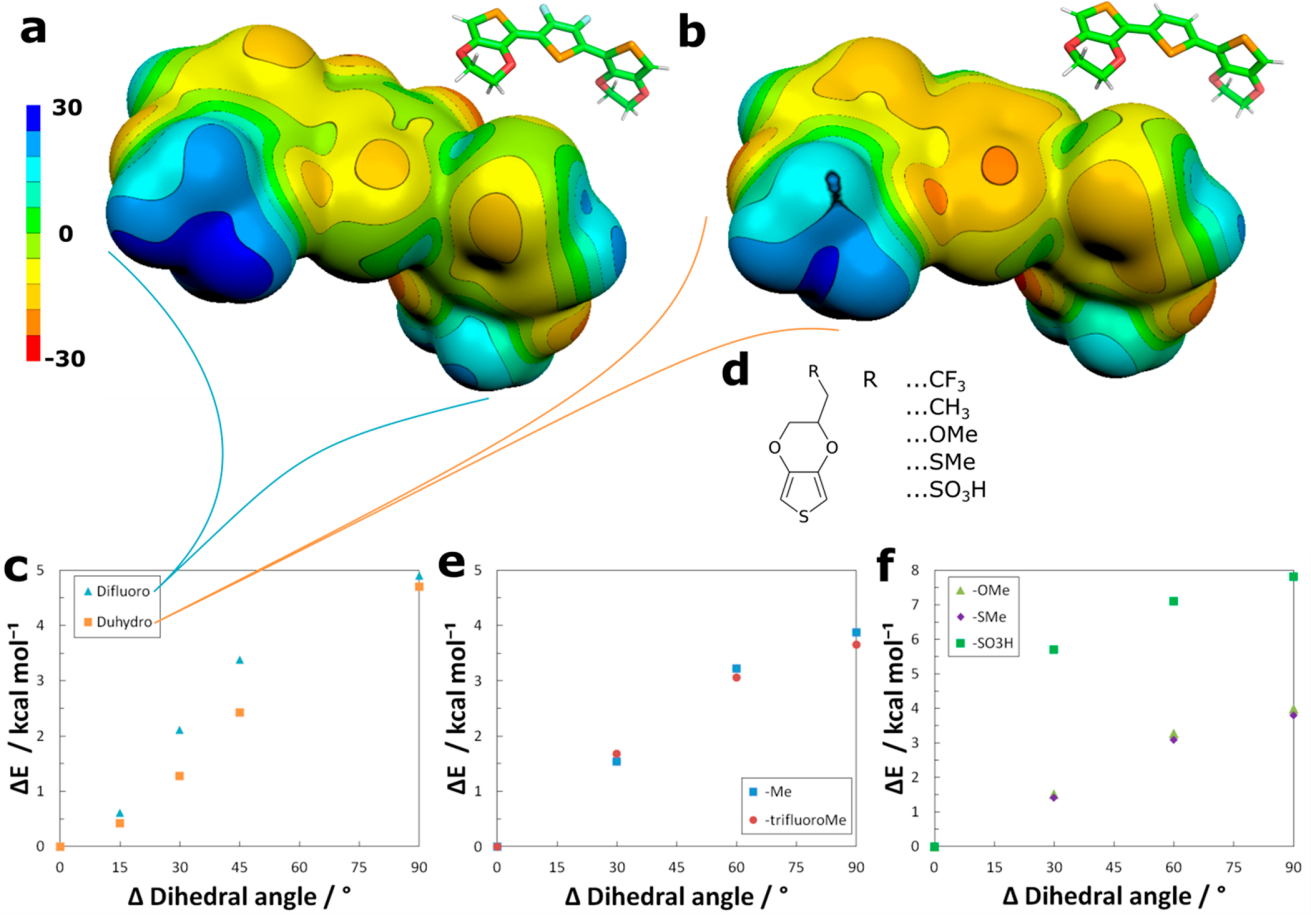
PEDOS analogues with a dioxane ring substituted for (a) hydrogen or (b) fluorine atoms (scale of color code in kcal mol^–1^). One monomer has been rotated by 90° so that its σ-hole can be surveyed. (c) Change of system energy upon rotation from the planar conformation. (d–f) Chemical structures of the monomers (d) and the development of their rotational energies upon rotation of their dihedral angle (e and f).

For the first case, we prepared additional model systems, in which we exchanged the central PEDOS-monomer by thiophene (electron-pushing) or 3,4-difluorothiophene (electron-withdrawing) (Figure 6 a-c). The chalcogen-bond should be thus weakened or improved, respectively.

The electron-withdrawing fluorine-substituents result in bigger (more positive) σ-holes than hydrogen-substituted ones (Figure 6a,b).

Consequently, the energy cost of rotating a monomer from a planar minimum was increased for the fluorine substituted system by enhancing the strength of the σ-hole lock (Figure 6c).

The respective band gaps were comparable, however, the fluorine-substituted species showed a slightly higher band gap relative to the hydrogen-substituted one (Figure.S6).

To demonstrate substitution effects on the σ-hole donor, we picked structures currently investigated in the scope of self-doped polymers.^35,57^ We specifically focused on the highly conductive structures of self-doped PEDOT published by Yano et al. (Figure 6d). There, the dopant is linked to the main system via a long alkyl-chain through an ether-bond, the effects of which we were particularly interested in. For simplicity, we replaced the complete ether-chain for methyl ether, thiomethyl ether, and sulfonate and observed the effects.

First, we started with introducing electro-negative trifluoro-methyl and compared its effect to a methyl group (Figure 6e). The substitution effects encountered were limited and below the accuracy of the method.^42,43^ Consequently, any conclusions from these results require experimental data for completeness. Since recent developments in high-performance conducting polymers clearly crave any form of improvement, we still proceed with their presentation.^22,58^

The electron-pushing substituent was favored by 0.3 kcal mol^–1^. Even relative to unsubstituted PEDOT, this translates to an increased rotational barrier of 0.2 kcal mol^–1^.

We were further interested in the substituents that have not only an inductive effect on the σ-hole donor, but also a resonance effect due to the free electron pairs. Consequently, we compared the difference between an ether and a thioether. For completeness, we also introduced the dopant directly instead of the ether to observe its effects.

Also, here, the difference was below the limit of the accuracy of the mehtod, which confirms that the position of the ether was well chosen to have minimal influence (Figure 6f).^35^ Yet, also here, the difference was in favor of the ether over the thioether, and a rotational barrier of +0.2 kcal mol^–1^ was observed. The resonance effect of the free-electron pairs in oxygen outweighs its inductive (electron-withdrawing) effect. For a clearer picture and to overcome the error of the method, a recalculation via CCSD would be advisible.

Since we were interested in illustrating only the effects of the substituent on the chalcogen bond, we were limited in the size of the side chain; consequently, we omitted selenium and tellurium substitution.

The side-chain interaction becomes apparent in the case of direct substitution via the dopant in the case of −SO_3_H (Figure 6d). Here, the planar conformation is substantially stabilized, and a steep increase is observed after rotation from 0 to 30° (6 kcal mol^–1^). At closer investigation, however, this substantial stabilization stems from hydrogen-bonding between the sulfonate substituents (Figure S7). Albeit, this certainly helps planarization and consequently conductivity; this was not entirely reflected by experimental results.

Occams razor suggests that said hydrogen bonding is a double-edged sword: it does not simply foster planarity, it causes strong interactions: it creates a strong energy barrier between the planar and nonplanar species; once on one side of the barrier, surpassing it by a postprocessing method becomes overly challenging. As a consequence, limited conductivities are achieved, in particular, where long alkyl-chains are allowed for a higher degree of freedom.

This potentially self-doped species presented us with the opportunity to test these functionals applicability to simulate doped states. We calculated the undoped (singlet) and self-doped charged species (doublet) and observed the emergence of states as previously done by Zozoulenko et al. for PEDOT (SI, Figure S8).^38^ From singlet to doublet, a shift in band gap is observed, with the gap growing larger upon doping (2.0 to 2.2 eV). Our findings agree with the literature that a single, polaron state emerges upon doping at 0.5 eV above the valence band or HOMO level. A more in-depth investigation would be beyond the scope of this work.

We hypothesize that the tremendous improvement in conductivity by Yano et al.^35^ may be related to the introduction of a branched alkyl chain. The methyl group next to the doping dopant possibly acts as a steric interference in the H-bond and correlates with the conductivity improvement by 2 orders of magnitude.^57^ This limits the formation of unwanted interactions and consequently improves charge-transport. To answer this question in sufficient detail is beyond the scope of this paper.

## Conclusions

The performed quantum mechanical analysis of PEDOT analogues with the ωB97X-V functional demonstrates its applicability and merit with regards to systems that unite extended conjugation and chalcogen bonds. Consequently, we find a useful tool for future studies, as this method delivers reliable results at a quarter of the computational time. A particularly good match with experimental results for band gaps was found. Band gaps predicted for PEDOT suggest that it might become possible to correlate experimental and theoretical results to gain insight with regards to the level of disorder present in materials. In general, the ωB97X-V functional allows the efficient study of large systems such as dodecamers, which correspond to the chain length typical for PEDOT.^19,29^

We find that oxygen in the case of PEDOF is small enough to avoid repulsion in the *trans*-planar (0°) conformation. Substitution by larger chalcogens in the dioxane group will well lead to adverse effects.

We present a possible explanation for how a planar PEDTT may have been achieved experimentally and attribute this to a strong, noncovalent interaction with the dopand.^36^

Finally, we demonstrated the concept of chalcogen bond tuning in the design of conductive polymers by modulation of the σ-hole and tuning of the σ-hole acceptor, respectively. The chalcogen bonds were enforced upon the introduction of electron-withdrawing groups close to the σ-hole, whereas the latter improved in the presence of polarizing substituents with free-electron pairs such as ethers. Applied well, this may have relevant consequences for the emerging field of highly conductive, (self-doped) conductive polymers.^19,29,35,36,57,58^

## Supporting Information Available

A list of local minima, including their dihedral angle and stability; bond-length analysis of global minima; image of the helical conformation of PEDTT-dodecamer; band gap development of angular scans calculated by ωB97X-V functional (oxygen and sulfur series, σ-hole tuning); angular scan results (energy and band gap) calculated by B3LYP functional; band gap development of angular scans calculated by ωB97X-V functional; illustration of polar contacts in PEDOT directly substituted by its dopand (SO_3_H) (PDF)
