# Supplementary material for: Strategies for the Design of PEDOT Analogues Unraveled: the Use of Chalcogen Bonds and σ-Holes
Source: J Phys Chem A. 2023 Apr 19;127(17):3779–87. doi: 10.1021/acs.jpca.2c08965 (PMC10165655; doi:10.1021/acs.jpca.2c08965)
Supplement: Supplementary file 1 — jp2c08965_si_001.pdf [file jp2c08965_si_001.pdf]

Supporting information: Strategies for the Design of PEDOT Analogues Unravelled: the Use of Chalcogen Bonds and σ-Holes

Dominik Farka*,+, Kristian Kříž+, Jindřich Fanfrlík
*Corresponding author
+these authors contributed equally to the manuscript

Local Minima

For PEDOF, PEDOT and PEDOS the global minimum was of planar conformation, for PEDTF it was near-planar. For PEDTT and PEDTS the minima were such that the chalcogen in the aromatic ring would interact attractively with the chalcogen of the neighbouring monomers thiadioxane ring; i.e. such that σ-hole and lone-pair were facing each other.

Table 1 describes characteristics of products of optimization, which lie higher in energy than minimum structures.

In local minima of PEDOF, PEDOT2 and PEDOS2 the five-membered-ring chalcogens are distorted by about 150° from the planar conformation ("cis" conformation).

Table 1: The local minima. Energy difference from the minimum conformation ΔE is in kcal/mol (computed on the same level as conformation energies of angular scans), angles are in degree.

	PEDOF	PEDOT	PEDOT2	PEDOS	PEDOS 2	PEDTF
D1	159	88	45	89	48	48
D2	152	92	46	92	48	49
ΔE	0.8	7.6	4.6	11.8	8.9	4.4

	PEDTT	PEDTT2	PEDTT3	PEDTS
D1	76	86	63.0	81
D2	78	60	161.9	81
ΔE	0.4	0.2	0	2.4

Bond-Lenght Analysis

The bond lenghts of the conjuagated systems were narrowly distributed in all investigated systems (1.37-1.46 Å, Table. 1).[1] We estimate that the experimentally observable differences are mainly caused by the systems tendency to planarize.

It should be noted, that the length of the inter-monomer bond ("B4" in Fig. 4) correlates with the planar character in our model systems. Indeed, this measure is shorter for all planar systems and indicates predisposition for the quinoid form.

Table 2: Bond lengths in optimized structures. St. dev is standard deviation for bond lengths for all carbon-carbon bonds of the conjugated systems e.g. C-C bonds of five-membered rings and bond connecting these rings.}

	PEDOF	PEDOT	PEDOS	PEDTF	PEDTT	PEDTS
	planar	planar	planar	near-planar	distorted	distorted
			Bond length / Å			
B1	1.37	1.37	1.36	1.37	1.37	1.37
B2	1.43	1.42	1.43	1.44	1.44	1.44
B3	1.38	1.38	1.38	1.38	1.38	1.37
B4	1.43	1.44	1.43	1.43	1.46	1.45
Std. Dev.	0.027	0.02	0.031	0.031	0.037	0.039

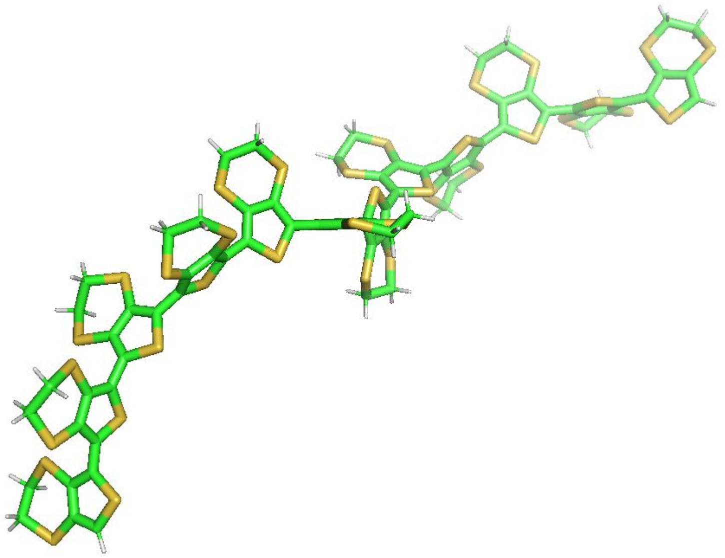

Figure S1: Dodecamer of PEDTT optimized into a helical conformation.

Angular Scans

We recalculated the ωB97X-V -results which reportedly gives improved results for σ-holes and repulsions alike [2,3] with the B3LYP to gain comparability with literature values.[4] Comparable results regarding the position of energetical minima and local maxima were obtained. In the case of PEDTT, the repulsion in the planar-conformation was overestimated by a factor of two in the case of B3LYP compared to the ωB97X-V, while the interactions in the "cis"-conformation are in agreement (Fig.S3).

The relevant bandgaps for ωB97X-V and B3LYP are given in Fig. S2 and S3, respectively. The discussion of these results is found in the main manuscript.

The evolution of the bandgap of the fluorinated and hydrogenated species (main text "Substitution Effects on the Chalcogen Bonds") is given in Fig. S4.

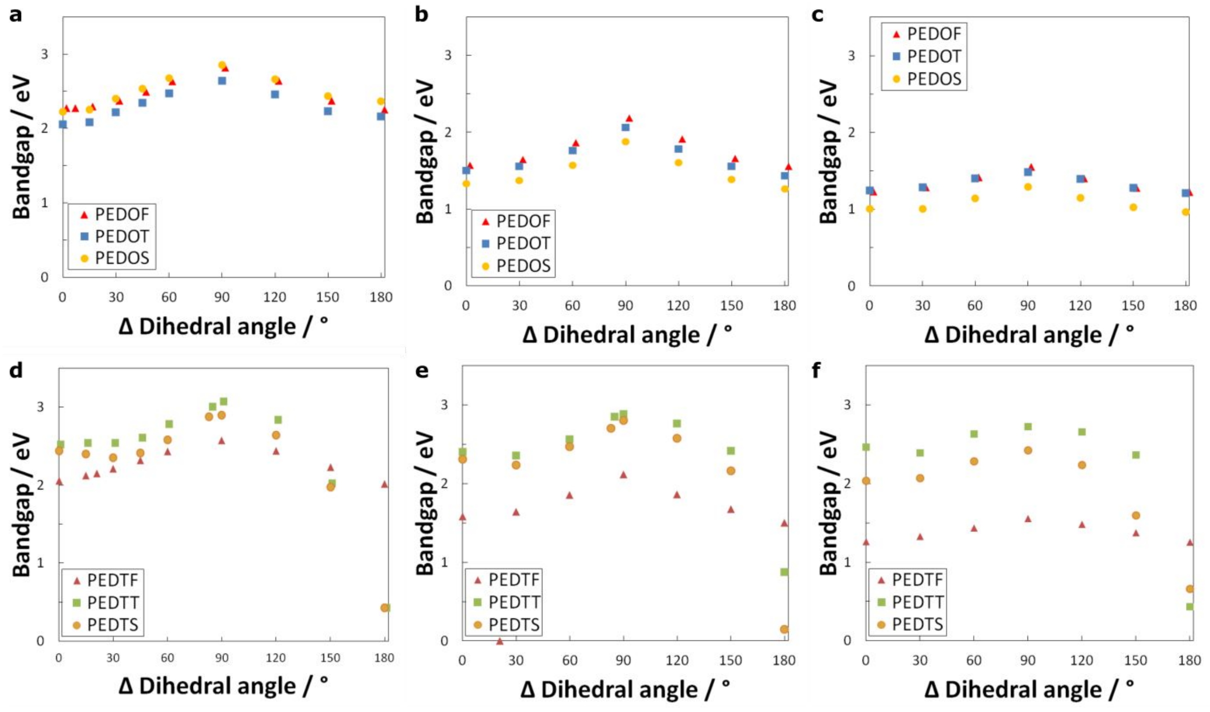

Figure S2: Badgap evolution dependent on dihedral angle corresponding to the calculations shown in Fig.5 (ωB97X-V). A comparison between the trimers, hexamers, and dodecamers of the oxygen (**a-c**) and sulphur series (**d-f**).

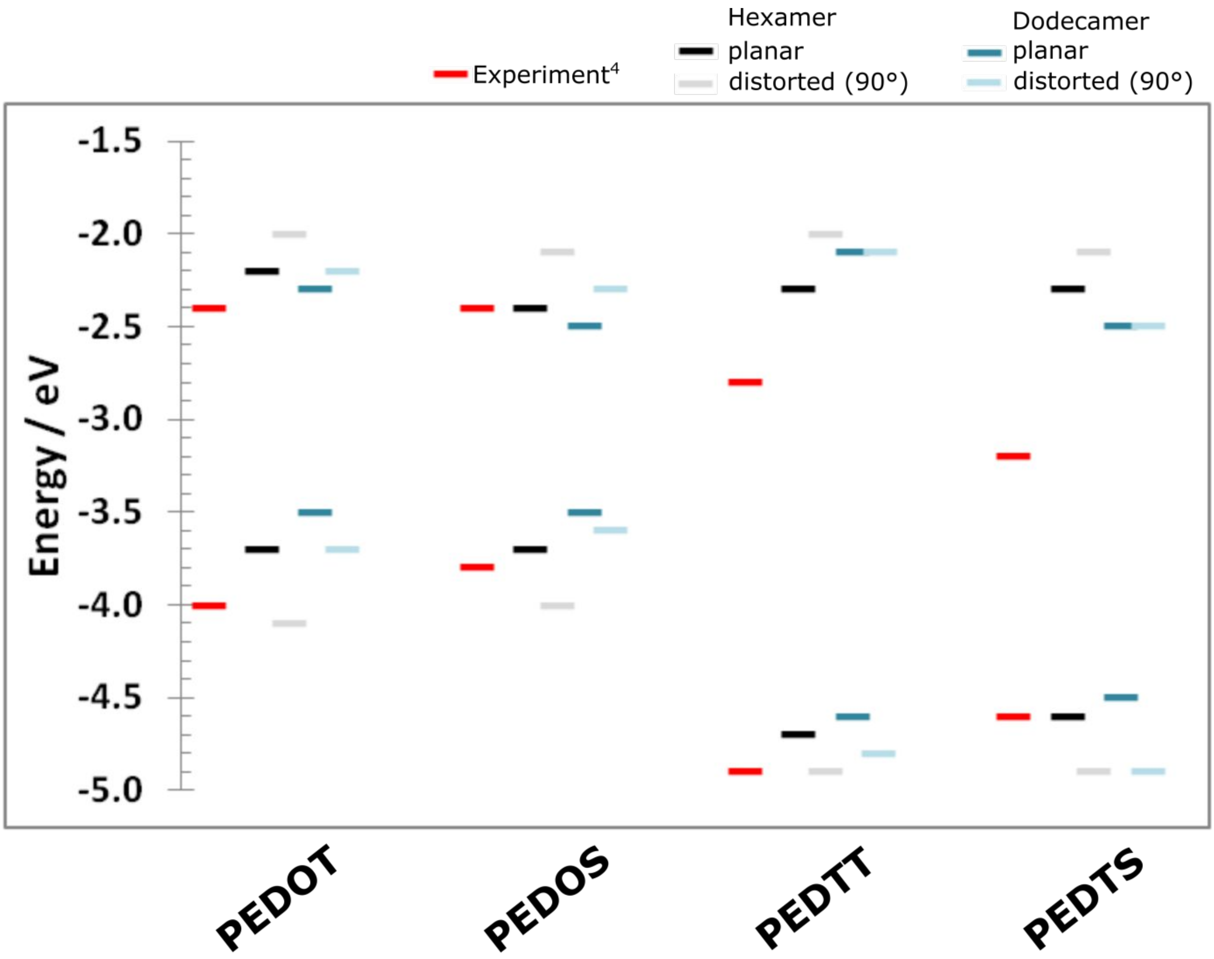

Figure S3: Band gaps of select polymers depicted as the HOMO and LUMO (Fig.S2). Experimental values according to Wijsboom et al.[4] Also here, the computational (ωB97X-V) values planar of the planar hexamer and distorted dodecamer were closest to experimental results.

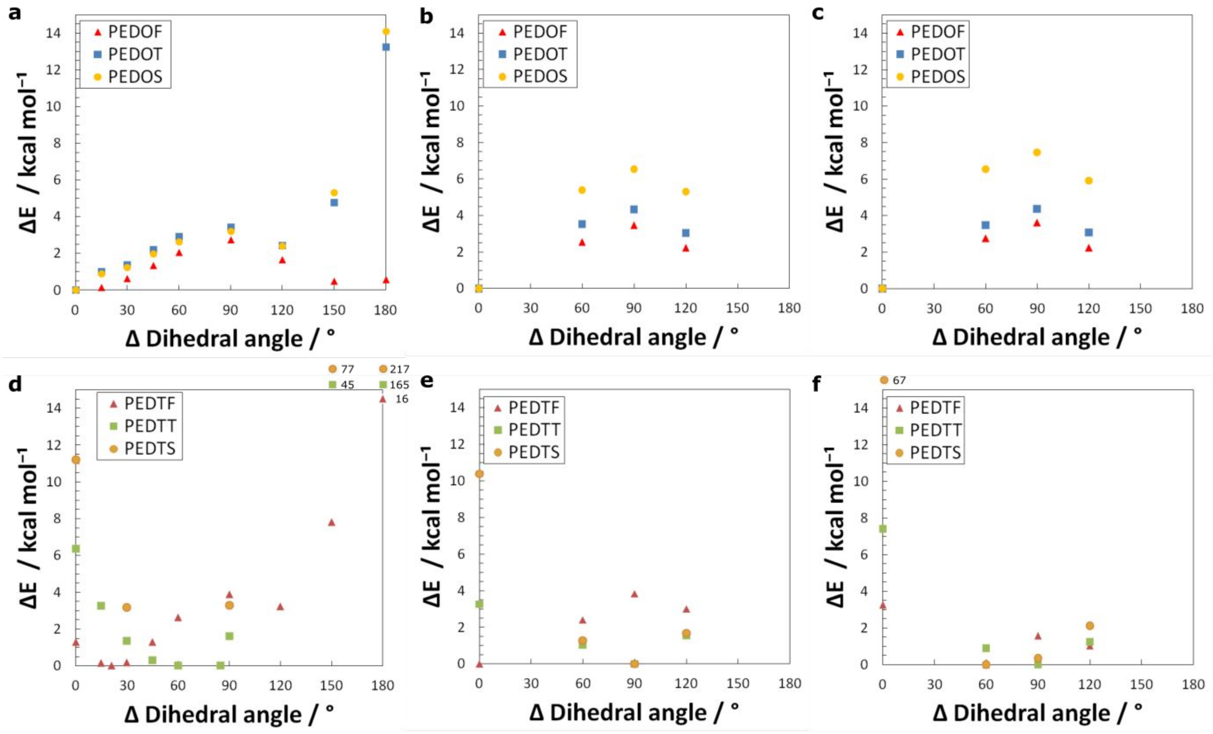

Figure S4: Conformational energies (ΔE) dependent on dihedral angle recalculated via B3LYP/D3BJ analogous to Fig.5. A comparison between the trimers, hexamers, and dodecamers of the oxygen (**a-c**) and sulphur series (**d-f**).

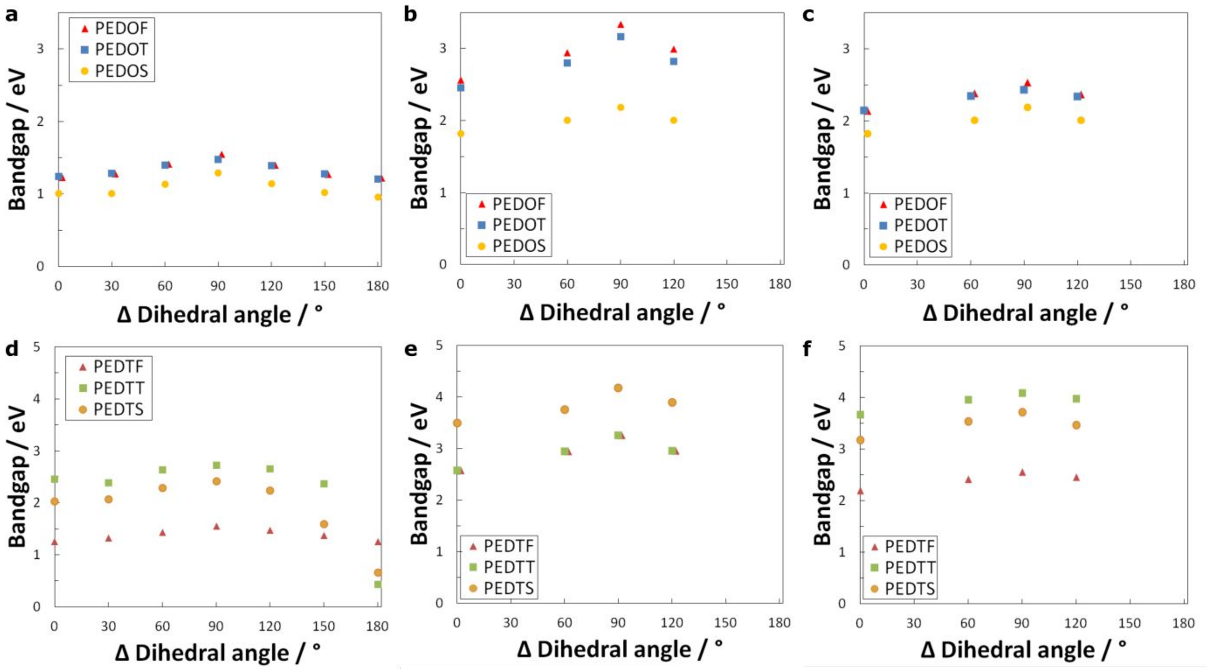

Figure S5: Bandgaps dependent on dihedral angle corresponding to Fig. S2 (B3LYP/D3BJ). A comparison between the trimers, hexamers, and dodecamers of the oxygen (**a-c**) and sulphur series (**d-f**).

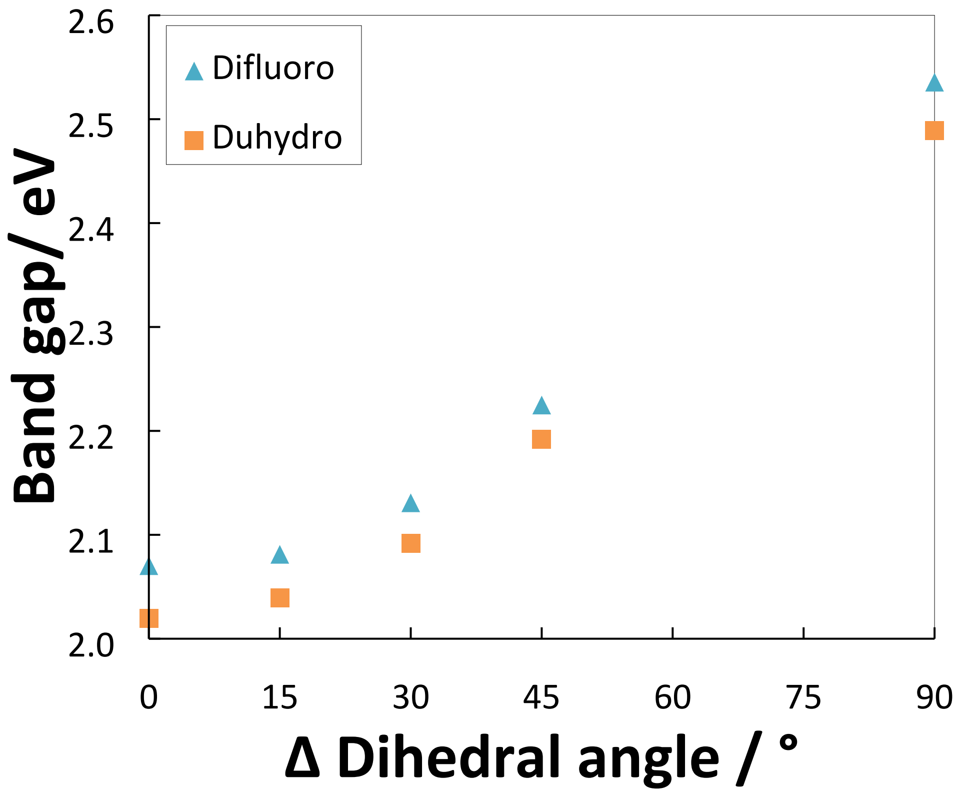

Figure S6: Evolution of the bandgap of trimers depicted in Fig.6a,b (wB97X-V/def2-DZVP). Fluorination of the selenophene-ring in the 3,4 position lead to a marginal increase in band gap.

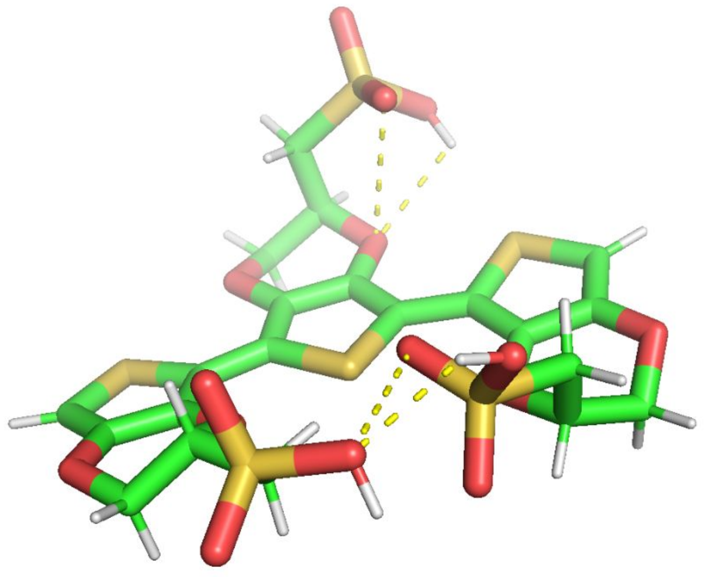

Figure S7: Illustration of polar contacts forming in the case of PEDOT directly substituted with the dopant (-SO_3H). The hydrogen-bond between the polar substituents leads to a substantial stabilization of the planar conformation. Image generated by Pymol software.

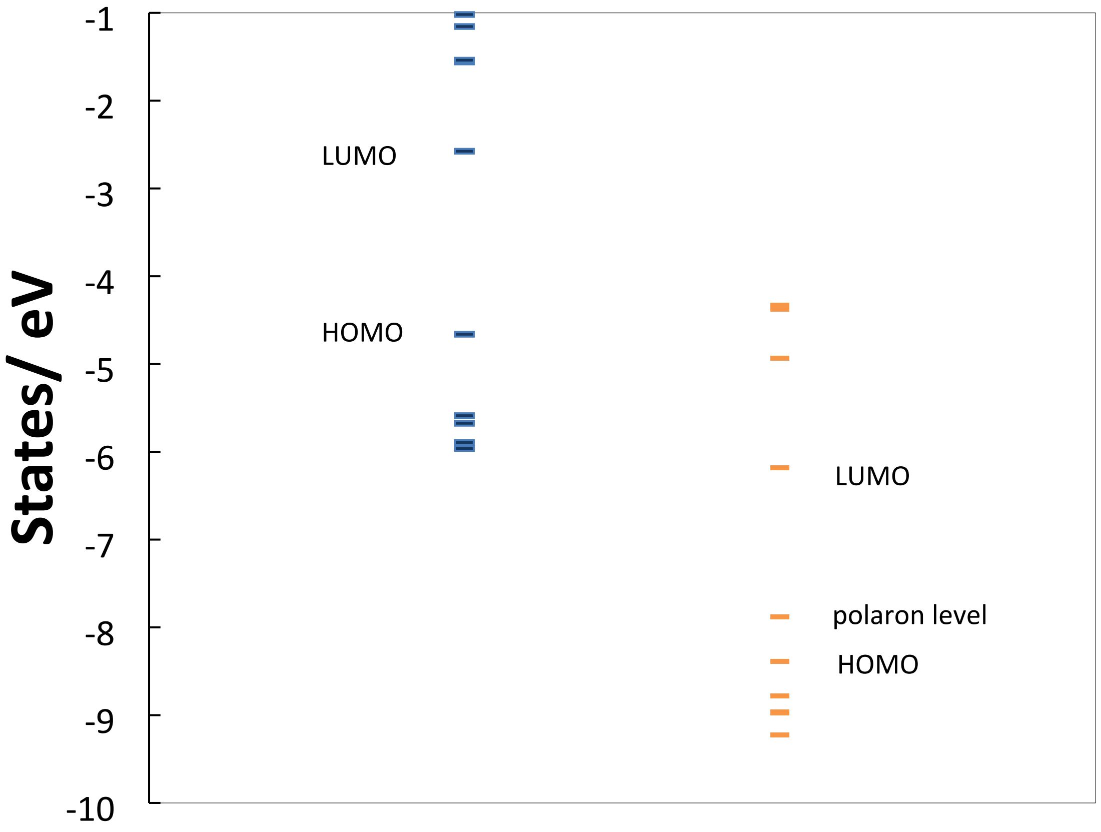

Figure S8: Electronic structure of PEDOT-sulphate trimer-chains in undoped singlet state (left, blue), and self-doped doublet (right, orange) radical state. We agree with results by Zozoulenko et al. regarding the emergence of a single polaron level in the gap upon doping. The HOMO-LUMO level rises from 2.0 eV to 2.2 eV upond doping, whereas the gap towards the polaron state is equivalent to 0.5 eV.

Literature

(1) CRC Handbook of Chemistry and Physics. In *Section 5: Thermochemistry,Kinetics, Electrochemistry, and Solutionchemistry*; John R. Rumble, Ed.; Taylor & Francis Group.

(2) Kříž, K.; Řezáč, J. Non-Covalent Interactions Atlas Benchmark Data Sets 4: σ-Hole Interactions. *Phys. Chem. Chem. Phys.* **2022**, *24* (24), 14794–14804. https://doi.org/10.1039/D2CP01600A.

(3) Kříž, K.; Nováček, M.; Řezáč, J. Non-Covalent Interactions Atlas Benchmark Data Sets 3: Repulsive Contacts. *J. Chem. Theory Comput.* **2021**, *17* (3), 1548–1561. https://doi.org/10.1021/acs.jctc.0c01341.

(4) Wijsboom, Y. H.; Sheynin, Y.; Patra, A.; Zamoshchik, N.; Vardimon, R.; Leitus, G.; Bendikov, M. Tuning of Electronic Properties and Rigidity in PEDOT Analogs. *J. Mater. Chem.* **2011**, *21* (5), 1368–1372. https://doi.org/10.1039/C0JM02679D.
